# Dynamic thresholding and robust contrastive techniques for enhanced semi-supervised cardiac segmentation

**DOI:** 10.1371/journal.pone.0342567

**Published:** 2026-04-06

**Authors:** Yafei Mi, Jie Zhang, Hui Jin, Jiayi Yin, Yuyang He, Guojie Xie, Shiqi Yan, Yongping Fu

**Affiliations:** 1 Department of Cardiology, Taizhou Hospital of Zhejiang Province affiliated to Wenzhou Medical University, Linhai, Zhejiang, China; 2 Cardiovascular Disease Clinical Translational Innovation Center of Shaoxing University, Shaoxing, Zhejiang, China; 3 Innovation Centre for Information, Binjiang Institute of Zhejiang University, Hangzhou, Zhejiang, China; 4 Department of Cardiology, Linhai Second People’s Hospital, Linhai, Zhejiang, China; 5 Digital Zhejiang Technology Operation Co., Ltd., Hangzhou, Zhejiang, China; 6 Department of Cardiovascular Medicine, Affiliated Hospital of Shaoxing University, Shaoxing, Zhejiang, China; University of Salerno: Universita degli Studi di Salerno, ITALY

## Abstract

Cardiac segmentation plays a crucial role in the diagnosis of cardiovascular diseases. However, the manual annotation of cardiac structures is a labor-intensive and time-consuming task that requires highly trained experts. Moreover, the availability of labeled data for training segmentation models is often limited due to the challenges associated with acquiring accurate annotations. To address this issue, we propose a novel semi-supervised cardiac segmentation framework that only needs a small set of labeled data with a larger pool of unlabeled data. We propose three strategies: dynamic pseudo-label threshold map, robust entropy minimization and contrastive consistency from the perspective of pseudo-labeling, entropy minimization and consistency regularization. Specifically, we propose a pixel-wise, class-wise and adaptive map to generate threshold maps and use the map for robust entropy minimization to reduce the noise from low-confidence samples. Besides, to utilize the unlabeled data sufficiently, we add contrastive consistency loss to implement regularization. Extensive experiments on the ACDC and MMWHS datasets demonstrate that our method achieves competitive performance compared to state-of-the-art approaches across various labeled data ratios. Ablation studies further validate the effectiveness and robustness of each component. Our framework shows strong potential for accurate diagnosis with limited annotations, and our code will be made publicly available.

## 1 Introduction

Cardiac segmentation plays a crucial role in medical imaging analysis, specifically in the field of cardiology. It involves the accurate delineation of the heart’s anatomical structures from medical images, such as magnetic resonance imaging (MRI) or computed tomography (CT) scans. Accurate segmentation enables the planning of surgical interventions, such as valve replacements or cardiac resynchronization therapy.

In recent years, deep learning techniques have shown great potential in automating cardiac segmentation tasks [1–5]. However, the success of these deep learning methods is heavily predicated on the availability of large volumes of meticulously annotated data. In the context of medical imaging, acquiring such extensive labeled datasets presents significant challenges due to the high cost, the requirement for specialized domain-specific expertise from cardiologists and radiologists, and the protracted, laborious process involved in generating accurate, pixel-level annotations [6]. This scarcity of labeled data severely limits the applicability and generalization capabilities of fully supervised deep learning models in real-world clinical settings, where diverse patient populations, varying anatomical structures, and differing imaging protocols are common.

To circumvent this critical data bottleneck, many researchers have explored annotation-efficient methods for medical image segmentation, including data augmentation [7,8], utilizing external related datasets [9], and particularly, leveraging unlabeled data through semi-supervised learning [10–15]. Among these, semi-supervised segmentation has emerged as a highly practical and impactful approach [16], promoting the synergistic utilization of readily obtainable unlabeled data in combination with a limited quantity of labeled data for training robust segmentation models. This technique holds significant promise for advancing clinical applications by enabling model deployment with less reliance on costly manual annotations.

However, despite its promise, existing semi-supervised segmentation methods face substantial hurdles, especially when applied to complex medical images such as cardiac scans [17]. Medical images often exhibit intricate anatomical structures, subtle pathological features critical for diagnosis, and considerable variations stemming from different imaging modalities, patient physiologies, and acquisition settings. These complexities make it exceptionally difficult for current semi-supervised methods to accurately estimate the uncertainty and confidence of generated pseudo-labels [12,16]. For instance, the often-fuzzy boundaries between cardiac chambers or the presence of small, irregular lesions can lead to ambiguous predictions, resulting in noisy or incorrect pseudo-labels that can mislead model training.

Furthermore, effectively and efficiently leveraging the vast amount of unlabeled data remains a challenge. Many existing methods [11,18,19] typically rely on calculating a fixed confidence threshold to select “high-quality" pseudo-labels for supervision. This static thresholding approach, however, is inherently problematic. It fails to adapt to the dynamic evolution of pseudo-label quality throughout the training process; what might be considered high-confidence early in training could still be quite noisy, while later, slightly lower-confidence predictions could be highly accurate. Consequently, a static threshold risks either discarding valuable information by being too conservative or introducing significant noise by being too permissive, thereby hindering optimal model performance. Beyond pseudo-label selection, many current methods primarily focus on pixel-level consistency [11], potentially underutilizing the rich contextual and structural information present in unlabeled medical images that could be exploited for more robust learning.

Based on the above critical observations and the identified limitations in current semi-supervised cardiac segmentation, we propose a novel semi-supervised segmentation framework designed to robustly leverage a small amount of annotated data alongside a large pool of unlabeled data to achieve superior outcomes. Specifically, to overcome the limitations of static thresholding and accurately estimate pseudo-label confidence, we introduce a dynamic pseudo-label threshold map strategy. This strategy generates a pixel-wise, class-wise, and adaptive map, enabling a more nuanced and accurate selection of high-confidence pseudo-labels throughout the dynamic training process. Furthermore, to mitigate the detrimental impact of low-confidence pseudo-labels and amplify the beneficial effect of high-confidence ones, we propose a robust entropy minimization loss function that explicitly leverages the insights from our dynamic threshold map. Lastly, to more effectively and efficiently utilize the rich information within unlabeled data beyond just pseudo-labeling, we introduce a novel contrastive consistency strategy that optimizes the model in an unsupervised and contrastive manner, capturing broader structural relationships. Extensive experiments are conducted on two cardiac segmentation datasets ACDC [20] and MMWHS [21]. Experimental results on two cardiac segmentation benchmarks show that our method delivers strong, competitive performance compared to current state-of-the-art approaches under various percentages of labeled data during training. We also conduct extensive experiments and detailed analysis to thoroughly evaluate the effectiveness and robustness of our method under various settings, including challenging low-data regimes.

Our main contributions can be summarized as follows:

- We propose a novel semi-supervised segmentation framework for cardiac segmentation, which only needs a small set of labeled data and can achieve competitive results as a fully supervised model.
- We propose dynamic pseudo-label threshold map, robust entropy minimization and contrastive consistency strategies. They can not only generate pixel-wise, class-wise and adaptive threshold maps but also utilize the unlabeled data efficiently and effectively.
- Our method demonstrates state-of-the-art competitive performance on two cardiac segmentation datasets, as evidenced by comprehensive benchmarking.

## 2 Related work

### 2.1 Cardiac image segmentation

Cardiac segmentation has garnered significant attention in the field of medical imaging analysis. In recent years, numerous studies have focused on developing robust and efficient techniques for cardiac segmentation. Early works [1,22–24] utilized deep fully convolutional neural network (FCN) [25] architectures to address cardiac MRI segmentation. For example, [22] proposed to tackle the problem of automated left and right ventricle segmentation through the application of a deep fully convolutional neural network architecture. [1] used an ensemble of UNet [26] inspired architectures for segmentation of cardiac structures on each time instance of the cardiac cycle. With the success of Transformer [27] in various fields, Transformer-based segmentation methods [2–5] have surpassed FCN-based methods. This is due to the intrinsic locality of convolution operations. Besides, U-Net generally demonstrates limitations in explicitly modeling long-range dependency. Therefore, [2] proposed TransUNet, which merits both Transformers and U-Net. TransUNet utilized Transformers as strong encoders to enhance finer details by recovering localized spatial information. Later, [4] proposed the Fully Convolutional Transformer (FCT) to learn fine-grained image representations and effectively capture long-term dependencies.

However, these methods all need large volumes of data to obtain desirable performance. It is difficult to acquire a large amount of annotated data, particularly for medical imaging where experts are needed to provide reliable and accurate annotations [28]. In contrast, our paper concentrates on semi-supervised segmentation where only a small amount of labeled data is needed and can still achieve desirable results.

### 2.2 Semi-supervised learning

Semi-supervised learning can be divided into five main groups [29]: generative methods, consistency regularization methods, graph-based methods, pseudo-labeling methods and hybrid methods. Generative methods [30–32] can the implicit features of data to better model data distributions. Consistency regularization methods [33,34] impose a regularization term to the loss function to specify the prior constraints. Graph-based methods [35–37] assume that a graph can be derived from the raw dataset, with each node representing a training sample and each edge indicating a measure of similarity between pairs of nodes. Pseudo-labeling [38,39] methods propose to select high-confident pseudo-labels and use these pseudo-labels for supervised training. Hybrid methods [40–42] combine the aforementioned methods to bring comprehensive performance improvement. For example, MixMatch [40] combines consistency regularization and entropy minimization into a unified loss function. FixMatch [42] combines consistency regularization and pseudo-labeling.

### 2.3 Semi-supervised medical image segmentation

Semi-supervised medical image segmentation can be divided into three main groups: pseudo-labeling methods [11,18], consistency regularization methods [13,43] and knowledge prior methods [44,45]. [11] propose a contrastive-radical network based on uncertainty estimation and a separate self-training strategy. [13] design a self-paced learning strategy for co-training that lets jointly-trained neural networks focus on easier-to-segment regions first, and then gradually consider harder ones. [44] propose a supervised local contrastive loss that leverages limited pixel-wise annotation to force pixels with the same label to gather around in the embedding space. [6] introduce a robust class-wise sampling method and dynamic stabilization for a better training strategy. [46] represent a specific type of data-adaptive regularization paradigm that aids in minimizing the overfitting of labeled data under high confidence values.

## 3 Proposed method

### 3.1 Problem formulation

In segmentation tasks, given an image $x\in R^{H\times W}$, the goal is to predict the label map $\hat{y}\in R^{H\times W\times C}$ where *C* is the class number. The value of $\hat{y}$ is between 0 and 1 representing the probability of the corresponding class. In semi-supervised segmentation, the datasets are divided into the labeled image set $D^{l}=\{(x_{i}^{l},y_{i}^{l}){\}}_{i=1}^{N_{l}}$ and unlabeled image set $D^{u}=\{x_{i}^{u}{\}}_{i=1}^{N_{u}}$ where *N*_*l*_ and *N*_*u*_ represent the number of images of the two image sets and $N_{l}\ll N_{u}$. The goal of semi-supervised segmentation is how to fully leverage the two image sets to get accurate predictions.

### 3.2 Overall architecture

**Fig 1:**
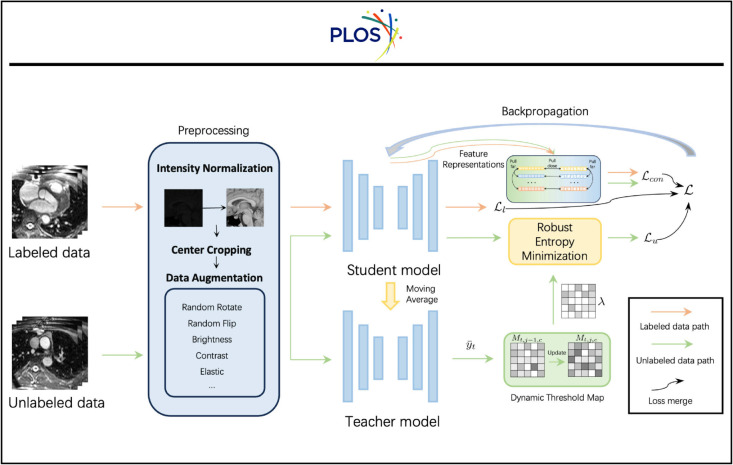
Overall architecture of our method.

Similar to other methods [6,47] in semi-supervised segmentation, we adopt the student and teacher setup [48] as the foundational structure for semi-supervised segmentation. Specifically, as shown in Fig 1, the teacher model with parameters $\hat{\theta }$ is updated by the exponential moving average of the parameters of the student model $\bar{\theta }$ as follows:

$${\hat{\theta }}_{t+1}=\beta {\hat{\theta }}_{t}+(1-\beta ){\bar{\theta }}_{t+1}$$ (1)

where *β* is the moving average coefficient [48] and *t* is the training step index. For the teacher model, we input the *x* from $D_{u}$ to obtain the pseudo-label $\bar{y}$ (see Sect 3.3 for details). For the student model, we input the *x* from $D_{l}$ and $D_{u}$ to get the prediction maps $\hat{y}$. For the labeled data $D^{l}=\{(x_{i}^{l},y_{i}^{l}){\}}_{i=1}^{N_{l}}$, we use the common supervised loss function:

$$L_{l}=\frac{1}{B}\sum_{i=1}^{B}L_{CE}({\hat{y}}_{i},y_{i}^{l})$$ (2)

where *B* is the batch size, $L_{CE}$ is the pixel-wise cross-entropy loss function. For the unlabeled data $D^{u}=\{x_{i}^{u}{\}}_{i=1}^{N_{u}}$, we use robust pixel-wise cross-entropy loss function $L_{RCE}$ (see Sect 3.4 for details):

$$L_{u}=\frac{1}{B}\sum_{i=1}^{B}L_{RCE}({\hat{y}}_{i},{\bar{y}}_{i})$$ (3)

where ${\bar{y}}_{i}$ is the pseudo-label for the *i*-th unlabeled sample generated by the teacher model.

Furthermore, to impose consistency regularization, we add a contrastive loss term $L_{con}$ (see Sect 3.5 for details). Therefore, our final loss function can be formulated as:

$$L=L_{l}+\lambda_{1}L_{u}+\lambda_{2}L_{con}$$ (4)

where $\lambda_{1}$ and $\lambda_{2}$ are the trade-offs between the losses. Then, we can use the loss function L to optimize the student model $\bar{\theta }$.

### 3.3 Dynamic pseudo-label threshold map

Generating pseudo-labels for self-training and entropy minimization is a common strategy in semi-supervised learning [11,18,19,42]. Empirically, not all samples are good enough to serve as pseudo-labels for self-training due to the noise in samples. When the low-quality pseudo-labels are used for self-training, the performance of the model will degrade. Existing semi-supervised segmentation methods often generate high-quality pseudo-labels by measuring the confidence of the predicted probabilistic distribution, which can be considered as a static threshold. However, this static threshold cannot accurately capture the evolving quality of labels during the dynamic training process.

To address the limitations of static thresholding, we propose a novel dynamic pseudo-label threshold map strategy. This strategy aims to generate a pixel-wise and adaptive confidence threshold map that evolves within each training epoch, updating batch-by-batch. This enables a more accurate and responsive selection of high-quality pseudo-labels for supervision.

Concretely, let *t* denote the current epoch and *k* denote the current batch index, where k∈{1,…,K} and *K* is the total number of batches per epoch. Let ${𝒳}_{t,k}^{u}=\{x_{1},\ldots ,x_{B}\}$ be the set of unlabeled images in the *k*-th batch of epoch *t*, with batch size *B*. For each image $x_{i}\in {𝒳}_{t,k}^{u}$, the teacher model generates a pseudo-label probability map ${\bar{y}}_{i}\in [0,1{]}^{H\times W\times C}$, where *H*, *W*, and *C* denote the height, width, and number of classes, respectively. We first compute the batch-averaged maximum confidence map ${\bar{M}}_{t,k}$ as:

$${\bar{M}}_{t,k}(h,w)=\frac{1}{B}\sum_{i=1}^{B}\max_{c=1}^{C}{\bar{y}}_{i}(h,w,c)$$ (5)

where (*h*,*w*) represents the spatial coordinates. The dynamic threshold map *M*_*t*,*k*_ is then updated using an Exponential Moving Average (EMA) to ensure temporal smoothness and stability across batches:

$$M_{t,k}(h,w)=\alpha M_{t,k-1}(h,w)+(1-\alpha ){\bar{M}}_{t,k}(h,w)$$ (6)

where α∈[0,1] is the momentum coefficient. For the initial state *M*_0,0_, we initialize all pixel values to 1/*C*, representing uniform uncertainty.

Furthermore, cardiac structures often exhibit significant class imbalance (e.g., ventricles are much larger than atria). To prevent smaller or lower-confidence classes from being ignored, we introduce a class-wise adaptation. We compute the class-specific confidence score $\epsilon_{t,c}$ for each class *c* within the current batch:

$$\epsilon_{t,c}=\frac{\sum_{i=1}^{B}\sum_{h,w}{𝕀}_{i,h,w}(c)\cdot \max_{c^{\prime }}{\bar{y}}_{i}(h,w,c^{\prime })}{\sum_{i=1}^{B}\sum_{h,w}{𝕀}_{i,h,w}(c)+\xi }$$ (7)

where ${𝕀}_{i,h,w}(c)=1[\arg\max_{c^{\prime }}{\bar{y}}_{i}(h,w,c^{\prime })=c]$ is the indicator function which equals 1 if the predicted class for pixel (*h*, *w*) in image *x*_*i*_ is *c*, and 0 otherwise. ξ is a small constant for numerical stability. We then normalize these scores to obtain the class-wise scaling factor $\eta_{t,c}$:

$$\eta_{t,c}=\frac{\epsilon_{t,c}}{\max_{c^{\prime }}\epsilon_{t,c^{\prime }}}$$ (8)

Finally, the class-wise dynamic pseudo-label threshold *M*_*t*,*k*,*c*_(*h*,*w*) is obtained by modulating the spatial threshold map:

$$M_{t,k,c}(h,w)=\eta_{t,c}\cdot M_{t,k}(h,w)$$ (9)

This mechanism ensures that classes with inherently lower prediction confidence (e.g., harder-to-segment small structures) are assigned proportionally lower thresholds, facilitating the recruitment of valid pseudo-labels for these classes.

For a given unlabeled sample, we generate the final confidence weighting map *γ* to select high-quality pixels for training. Let $c^{*}=\arg\max_{c}\bar{y}(h,w,c)$ denote the predicted class at location (*h*, *w*). A pixel (*h*, *w*) is considered reliable for its predicted class c^*^ if its predicted probability exceeds the corresponding dynamic threshold:

$$\gamma_{\left(h,w\right)}=\left\{ \begin{array}{cc} \bar{y}(h,w,c^{*}), & \text{if }\bar{y}(h,w,c^{*})>M_{t,k,c^{*}}(h,w)\\ 0, & \text{otherwise} \end{array}\right.$$ (10)

where $\gamma_{\left(h,w\right)}\in R^{H\times W}$ is the dynamic pixel-wise and class-wise confidence map (Table 1).

**Table 1:** Notation and definitions for important variables.

Symbol	Definition
*t*	Current training epoch index
*k*	Current batch index within epoch *t*
*B*	Batch size (number of images in a batch)
*C*	Total number of segmentation classes
*H*, *W*	Spatial height and width of the input image
(*h*, *w*)	Spatial coordinates of a pixel
*c*	Class index
*x*_*i*_	An input image from the unlabeled set ${𝒟}^{u}$
${\bar{y}}_{i}$	Probability map predicted by the teacher model for image *x*_*i*_
*M*_*t*,*k*_	The base dynamic threshold map for batch *k* in epoch *t*, computed via EMA
*α*	Decay factor for the Exponential Moving Average (EMA), α∈[0,1]
${𝕀}_{i,h,w}(c)$	Indicator function for pixel (*h*, *w*) of image *i* being classified as class *c*
$\epsilon_{t,c}$	The mean prediction confidence for class *c* within the current batch at epoch *t*
$\eta_{t,c}$	Class-wise scaling factor for class *c* in epoch *t*, normalized by the maximum mean confidence
*M*_*t*,*k*,*c*_	The final, class-adapted dynamic pseudo-label threshold for class *c* in batch *k* of epoch *t*
*γ*	Confidence map selecting high-quality pixels for the loss calculation

### 3.4 Robust entropy minimization

In Sect 3.3, we obtain a map *γ* that can dynamically measure the pixel-wise and class-wise confidence. For high-quality pixels, we wish they would contribute more to optimization while the influence of those low-quality pixels will be eliminated. Therefore, we propose a robust entropy minimization strategy by adding the confidence value *γ* to the cross-entropy loss function:

$$L_{RCE}=-\frac{1}{H\times W}\sum_{i=1}^{H\times W}\gamma_{i}p_{i}\log(p_{i})$$ (11)

where *i* indexes all pixel locations (*h*, *w*) in the image (flattened into a single dimension), $\gamma_{i}=\gamma_{\left(h,w\right)}$ is the corresponding confidence weight, and *p*_*i*_ is the confidence of the prediction at that location, which can be calculated as:

$$p_{i}=\max\left(\frac{\exp({\bar{y}}^{i}/\tau )}{\sum_{c=1}^{C}\exp({\bar{y}}_{c}^{i}/\tau )}\right)$$ (12)

where *τ* is a temperature hyperparameter. From Eq. 11, we can observe that the high-quality predictions (*i.e.* $\gamma_{i}$ is high) will contribute more to the loss function which in turn help the optimization process and the influence of low-quality predictions (*i.e.* $\gamma_{i}$ is small) will be alleviated.

### 3.5 Contrastive consistency

In Sects 3.3 and 3.4, we introduce two strategies for semi-supervised segmentation from the perspective of pseudo-labeling and entropy minimization. In this section, we will introduce a contrastive consistency regularization strategy.

A segmentation network F usually consists of an encoder E to project the image into a latent representation and a decoder D to reconstruct the representation into a segmentation map. Specifically, we input the images into the encoder to get the latent feature representation h=E(x). For a batch of images, we implement a series of image augmentation strategies, such as color jitter, random cropping, noise injection and cutout. Then, we will have two groups of representations h and $h^{\text{aug}}$ for original images and augmented images. Motivated by contrastive learning [49,50], we propose to make the representation of one image and its augmented image representation closer and push it far away from the representations of other images in the batch. We consider a modified *NT-Xent* loss [50] as our loss function. Let $\text{sim}(u,v)=u^{⊤}v/||u||||v||$ denote the dot product between $\ell_{2}$ normalized ***u*** and ***v*** (i.e. cosine similarity). The loss function for a positive example $\left(h_{i},h_{i}^{\text{aug}}\right)$ is as follows:

$$\ell_{h_{i},h_{i}^{\text{aug}}}=-\log\frac{\exp(\text{sim}(h_{i},h_{i}^{\text{aug}})/\tau )}{\sum_{t=1}^{B}1_{\left[t\neq i\right]}\exp(\text{sim}(h_{i},h_{t})/\tau )}$$ (13)

where *τ* is a temperature parameter, *B* is the batch size and $1_{\left[t\neq i\right]}\in \{0,1\}$ is a sign function evaluating to 1 if t≠i. Then the contrastive loss function can be formulated as:

$$L_{con}=\frac{1}{2B}\sum_{k=1}^{B}(\ell_{h_{k},h_{k}^{\text{aug}}}+\ell_{h_{k}^{\text{aug}},h_{k}})$$ (14)

where *B* is the batch size. Through contrastive consistency, the model can differentiate different samples and make consistent predictions for the same sample, which will improve the generalization ability of the model.

### 3.6 Synergistic interaction of components

The three core components of our framework work synergistically to enhance semi-supervised learning by addressing complementary aspects of the training process. The Dynamic Pseudo-label Threshold Map (DPTM) and Robust Entropy Minimization (REM) operate in the prediction space to ensure the reliability of pseudo-labels: DPTM acts as an adaptive filter to select high-confidence pixels, while REM further mitigates the impact of noise by weighting the optimization based on pixel-wise confidence. Complementing this, the Contrastive Consistency (CC) module operates in the feature space, enhancing the model’s discriminative power and global structural consistency. Crucially, these modules form a mutually reinforcing feedback loop: the improved feature representations learned via CC lead to more accurate predictions, which in turn enable DPTM to generate more precise thresholds and high-quality pseudo-labels for subsequent training iterations. This virtuous cycle ensures that the model progressively refines itself, effectively leveraging both labeled and unlabeled data.

## 4 Results

### 4.1 Datasets

#### 4.1.1 ACDC dataset.

ACDC [20] contains data from 150 multi-equipments cine-MRI recordings with reference measurements and classification from two medical experts. For every patient, it has around 15 volumes covering the entire cardiac cycle and expert annotations for left and right ventricles and myocardium.

#### 4.1.2 MMWHS dataset.

MMWHS [21] consists of 20 cardiac MRI samples with expert annotations for seven structures: left and right ventricles, left and right atrium, pulmonary artery, myocardium, and aorta.

### 4.2 Evaluation metrics

Following previous works [2,5,51], we use Dice Similarity Score (DSC), Jaccard Score (Jaccard), 95% Hausdorff Distance (95HD) and Average Surface Distance (ASD) for evaluation. DSC is defined as the ratio of the overlap between the predicted contour A and the real contour B. HD is defined by the maximum between the directed average Hausdorff distance *h*(*A*, *B*) and its reverse direction *h*(*B*, *A*) where A and B represent the ground truth and predicted segmentation, respectively. From the definition, we know that a higher DSC and lower HD mean better performance.

### 4.3 Implementation details

Following previous works [6,52,53], we use 1.25%, 2.5%, and 10% labeled data from ACDC and 10%, 20%, and 40% labeled data from MMWHS. We use UNet [26] as our backbone. We implement data augmentations to enlarge the datasets and avoid overfitting. All the experiments are conducted on a Ubuntu desktop with RTX3090 GPUs. We use the SGD optimizer with an initial learning rate 1*e*–2, momentum 0.9 and weight decay 5*e*–4. A cosine learning rate scheduler is implemented to the optimizer with $\eta_{i}=1e-2$ and $\eta_{f}=1e-3$ for initial and final learning rates. The batch size is set to 16 by default.

For experimental setup, all volumetric data underwent consistent preprocessing, including per-volume intensity normalization and center-cropping for background removal. During training, we applied an extensive suite of data augmentations to both labeled and unlabeled data with specified ranges: random rotation ($\pm 30^{\circ }$), scaling (0.7–1.3), elastic deformations (σ=10), random flipping (prob=0.5), and brightness adjustments (0.7–1.3). For both ACDC and MMWHS, we follow the official training/validation/test splits provided by the challenge organizers. We do not use early stopping, and the final checkpoint at the last training epoch is used for evaluation. For our method, all experiments (including main comparisons and ablation studies) are repeated with three fixed random seeds, and we report the mean ± standard deviation; baseline results follow either the numbers reported in their original papers or our single-run re-implementations under the same experimental setting.

We compare our method with several baselines and recent state-of-the-art semi-supervised cardiac segmentation methods: Self train [54], Data aug [55], Context Restoration [56], Mixmatch [40], Global [50], Global+Local [52], SemiContrast [44], PCL [57], ACINet [6], SSCI [53], PatchCL [19], UA-MT [58], SS-Net [59], DC-Net [60], CauSSL [61], BCP [47], SDCL [17], Strong teacher [62], and Improved-UniMatch [63].

To ensure fair and reproducible comparisons, state-of-the-art baseline methods are either taken from the results reported in their original papers or re-implemented under identical experimental conditions with consistent training budgets and data splits as our method. All methods share the same U-Net backbone, SGD optimizer with identical learning rate schedules, and are evaluated using the same hardware and evaluation metrics to ensure apples-to-apples comparisons.

### 4.4 Quantitative results

**Fig 2:**
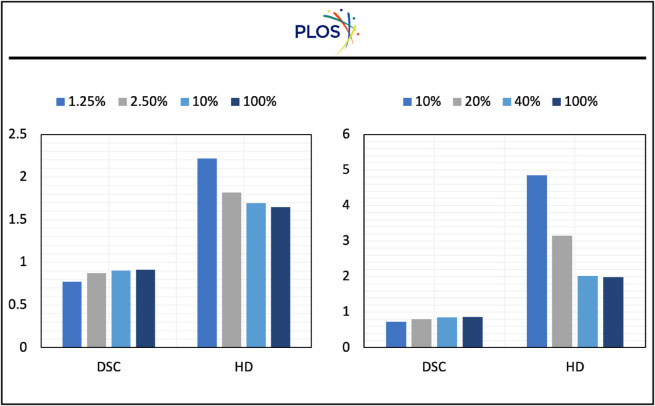
Quantitative evaluation of our proposed method on ACDC and MMWHS datasets under various labeled data ratios.

In Fig 2, we evaluate our proposed method on ACDC and MMWHS datasets under different percentages of labeled data during training. For comparison, we add a fully supervised training baseline which has 100% of labeled data. The results on the ACDC dataset are shown in Fig 2A. The DSC and HD of the model only using 10% data are 0.906 and 1.33, respectively while the DSC and HD of the fully supervised model are 0.915 and 1.645, respectively. We can observe that the performance of using only 10% of data is comparable to the fully supervised performance, which indicates the superiority of our methods. In Fig 2B, the DSC and HD of the model only using 40% data are 0.855 and 2.02, respectively while the DSC and HD of the fully supervised model are 0.863 and 1.986, respectively, where we can find similar conclusions. With a small portion of labeled data, our method can achieve competitive results as fully supervised training.

### 4.5 Comparison with state-of-the-arts

Table 2 presents the average DSC of different methods on the ACDC and MMWHS datasets under different percentages of labeled data during training. Overall, our proposed method achieves competitive or superior performance compared to other state-of-the-art methods on both datasets, with only a marginally lower DSC (–0.001) than SSCI on ACDC with a 10% of labeled data for training. Besides, we present a detailed comparison across different metrics on ACDC in Table 3. These results indicate that our method is highly competitive and often achieves the best performance across different settings.

**Table 2:** Comparison with state-of-the-art methods on ACDC and MMWHS datasets under different labeled data ratios. **Bold**: best result. Underline: second best result.

Method	Avg DSC (ACDC)			Avg DSC (MMWHS)		
	L=1.25%	L=2.5%	L=10%	L=10%	L=20%	L=40%
Self train [54]	0.713	0.848	0.873	0.532	0.642	0.798
Data aug [55]	0.727	0.784	0.855	0.531	0.663	0.782
Context restoration [56]	0.633	0.701	0.844	0.501	0.650	0.791
Mixmatch [40]	0.621	0.819	0.849	0.594	0.691	0.801
Global [50]	0.701	0.794	0.851	0.511	0.647	0.793
Global+Local [52]	0.757	0.826	0.886	0.617	0.710	0.794
SemiContrast [44]	0.574	0.738	0.836	0.497	0.637	0.759
PCL [57]	0.674	0.823	0.891	0.511	0.633	0.750
ACINet [6]	0.746	0.842	0.889	0.626	0.791	0.815
SSCI [53]	0.768	0.869	**0.907**	0.732	0.789	0.840
PatchCL [19]	0.759	0.857	0.894	0.715	0.784	0.844
SDCL [17]	0.770	0.863	0.898	0.728	0.790	0.839
Strong teacher [62]	0.761	0.841	0.854	0.731	0.778	0.845
Improved-UniMatch [63]	0.766	**0.877**	0.898	0.728	0.793	0.844
Ours	**0.775 ± 0.02**	0.874 ± 0.01	0.906 ± 0.01	**0.735 ± 0.02**	**0.803 ± 0.02**	**0.855 ± 0.01**

**Table 3:** Detailed comparison with state-of-the-art methods across various metrics on ACDC with a labeled data ratio of 10%.

Method	DSC(↑)	Jaccard(↑)	95HD(↓)	ASD(↓)
UA-MT [58]	0.817	0.706	6.88	2.02
SS-Net [59]	0.868	0.777	6.07	1.40
DC-Net [60]	0.894	0.814	1.28	0.38
CauSSL [61]	0.897	0.818	3.67	0.93
BCP [47]	0.888	0.806	3.98	1.17
Ours	**0.906 ± 0.01**	**0.841 ± 0.01**	**1.33 ± 0.25**	**0.33 ± 0.03**

**Fig 3:**
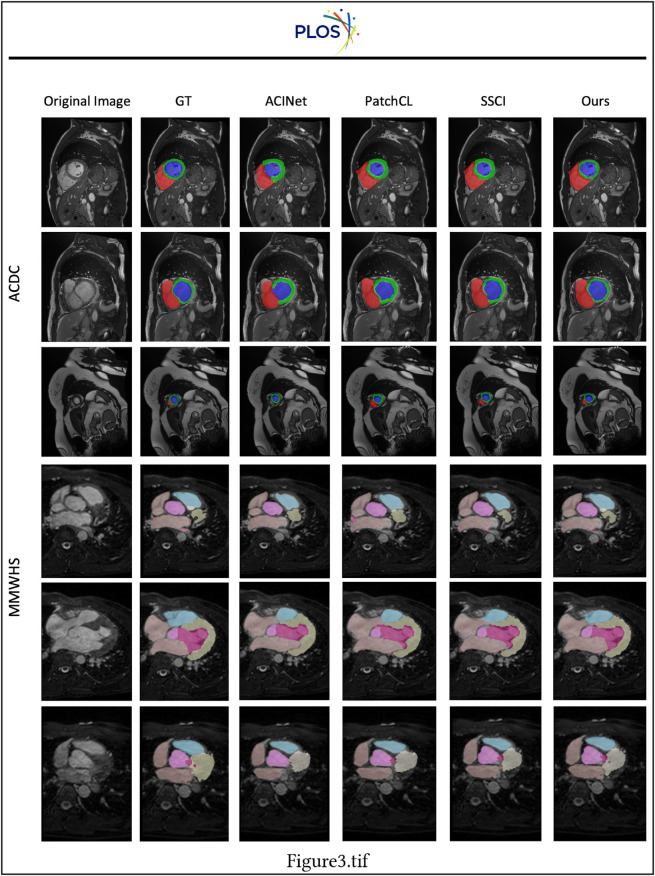
Qualitative comparison of different methods on the ACDC and MMWHS datasets. For ACDC, we use 2.5% labeled data. For MMWHS, we use 20% labeled data.

In Fig 3, we select three baselines with good performances (ACINet, PatchCL and SSCI) from Table 2 to visually compare and assess their performance. Observing the six instances presented in the figure, it is apparent that our method exhibits a significant advantage over other approaches, particularly in scenarios where the boundary textures are unclear and the segmented regions are small. For example, in the third row, our method can accurately segment the red region, whereas the other baseline methods fail to do so. This superiority is further demonstrated in the sixth row, where our method successfully segments the small red region which is surrounded by the yellow region, whereas the other three baselines fail to detect it. Besides, in the first and second rows, the compared methods produce thicker segmentations of the green circle region, whereas our method generates results that are more similar to the ground truth. The above observations further validate the effectiveness and superiority of our method.

### 4.6 Dynamic pseudo-label threshold map

Pseudo-label-based methods are very popular in semi-supervised segmentation [11,18,19]. However, different from previous methods, our method proposes a dynamic pseudo-label threshold map which is changing adaptively during the training process. Moreover, our dynamic pseudo-label threshold map can measure pixel-wise and class-wise confidence and can provide more accurate results. In Table 5, we present the comparison of performance between our dynamic pseudo-label threshold map strategy with recent pseudo-label-based methods. From the table, we can observe that our pseudo-label strategy outperforms other recent state-of-the-art pseudo-label strategies. Particularly, we can find that when we use less labeled data, the more the performance of our proposed method is ahead of the other pseudo-label strategies. This demonstrates the superiority of our dynamic pseudo-label strategy which can provide pixel-wise, class-wise and adaptive threshold maps.

**Fig 4:**
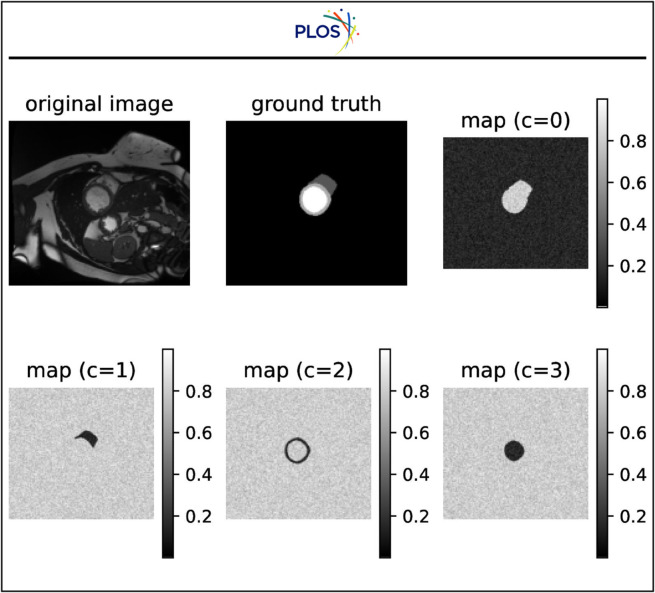
Visualization of dynamic pseudo-label threshold maps of an example in the ACDC dataset. *c* represents the class.

Furthermore, we visualize our dynamic pseudo-label threshold map in Fig 4 for a better understanding. We visualize an example in the ACDC dataset. There are four classes c=0,1,2,3 representing the background, RV, Myo and LV, respectively. The values in the map represent the threshold of the corresponding class. From the figure, we can observe that our threshold map can measure the confidence of the corresponding class accurately, demonstrating the effectiveness of our dynamic pseudo-label threshold map.

### 4.7 Per-structure analysis and model interpretability

To address the need for granular performance evaluation and assess the stability of our method, Table 4 presents the per-structure Dice similarity coefficient (DSC) and 95% Hausdorff Distance (95HD) with standard deviations across three independent runs. The results indicate that our method achieves balanced segmentation performance across the Right Ventricle (RV), Myocardium (Myo), and Left Ventricle (LV). Notably, the low standard deviations demonstrate the robustness of our approach, even with limited labeled data. The improvements are particularly significant for the Myocardium, where boundary definition is often ambiguous and challenging for semi-supervised methods.

**Table 4:** Per-structure segmentation results on the ACDC dataset (2.5% labeled data). Results are reported as mean ± standard deviation over 3 runs. RV: Right Ventricle, Myo: Myocardium, LV: Left Ventricle.

Method	RV		Myo		LV	
	DSC(↑)	95HD(↓)	DSC(↑)	95HD(↓)	DSC(↑)	95HD(↓)
SSCI [53]	0.865 ± 0.12	2.04 ± 0.45	0.815 ± 0.09	2.15 ± 0.32	0.927 ± 0.06	1.45 ± 0.25
PatchCL [19]	0.855 ± 0.14	2.25 ± 0.51	0.805 ± 0.11	2.38 ± 0.38	0.911 ± 0.08	1.62 ± 0.29
Ours	**0.875 ± 0.10**	**1.85 ± 0.38**	**0.820 ± 0.08**	**1.92 ± 0.26**	**0.928 ± 0.05**	**1.21 ± 0.18**

**Table 5:** Comparison of performance between our dynamic pseudo-label threshold map (DPTM) strategy with recent pseudo-label-based methods. For a fair comparison, we only implement the pseudo-label part in these methods (denoted as †) and then use the pseudo-labels for self-training. We report the average DSC in the table. Bold: best result. Underline: second best result.

Method	Avg DSC (ACDC)			Avg DSC (MMWHS)		
	L=1.25%	L=2.5%	L=10%	L=10%	L=20%	L=40%
CoraNet† [11]	0.719	0.814	0.831	0.597	0.723	0.796
PLR† [18]	0.721	0.806	0.841	0.711	0.746	0.807
PatchCL† [19]	0.726	0.831	0.863	0.704	0.751	0.822
**DPTM**	**0.753 ± 0.03**	**0.848 ± 0.02**	**0.872 ± 0.02**	**0.725 ± 0.03**	**0.780 ± 0.03**	**0.831 ± 0.02**

**Fig 5:**
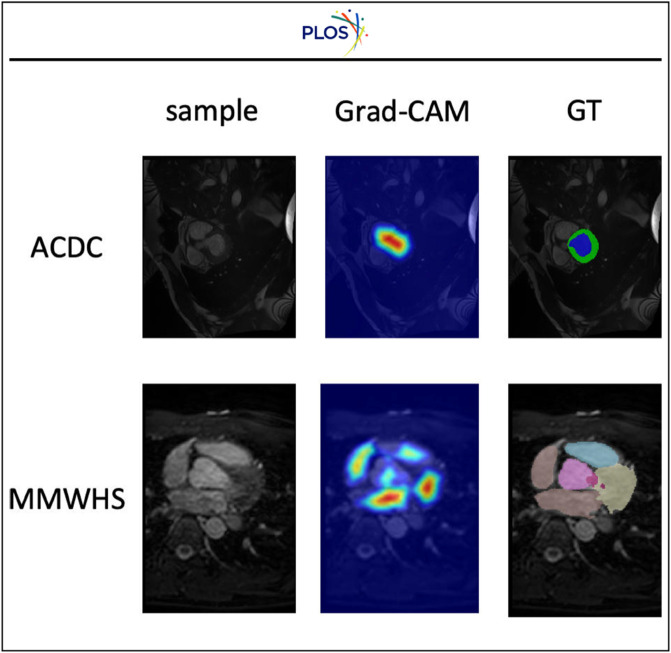
Grad-CAM visualization of the proposed method on the ACDC and MMWHS datasets. The top row shows the failure results on the ACDC dataset and the bottom row shows the successful results on the MMWHS dataset.

Furthermore, to investigate model behavior and analyze failure cases, we utilized Explainable AI (XAI) techniques. Fig 5 displays Grad-CAM visualizations for both failure and successful segmentation scenarios. The attention maps reveal that in successful cases (bottom row), the model focuses intensely on the specific cardiac structures, indicating that our Contrastive Consistency strategy effectively drives the network to learn discriminative features. In the failure case (top row), the attention is more diffuse or focused on incorrect boundary regions, highlighting the difficulty of segmenting low-contrast areas. This interpretability allows us to understand that while the model is generally robust, it can still struggle with extremely ambiguous boundaries.

Regarding predictive confidence and calibration, our Dynamic Pseudo-label Threshold Map (DPTM) implicitly functions as an uncertainty management mechanism. Instead of using a static threshold which may include poorly calibrated predictions, DPTM adapts to the learning state of the model class-wise. This effectively filters out high-uncertainty predictions from the pseudo-labeling process, ensuring that the model is trained primarily on reliable, well-calibrated predictions.

### 4.8 Ablation study

For a better understanding of our proposed method, we conduct a series of ablation experiments. The experiments can be divided into the effectiveness of the three main components of our method and loss trade-off terms.

#### 4.8.1 Component analysis.

In Table 6, we provide a comprehensive overview of the ablation experiments conducted on the three proposed components. The results clearly demonstrate the positive impact of each component on the model’s performance. Notably, our dynamic pseudo-label threshold map strategy emerges as the most influential factor, significantly enhancing the model’s performance. Additionally, the robust entropy loss contributes as the second most important factor in improving the model’s performance. Besides, the synergy achieved by combining these three components yields consistent and substantial improvements in the model’s performance. These findings confirm the effectiveness and significance of our proposed components in augmenting the model’s capabilities.

**Table 6:** Benefits of our proposed module dynamic pseudo-label threshold map (DPTM), robust entropy minimization (REM), and contrastive consistency (CC). For ACDC, we use 10% labeled data. For MMWHS, we use 40% labeled data. † denotes the best performance when attached with only one component and * represents the best results attached with two components.

DPTM	REM	CC	ACDC		MMWHS	
			DSC(↑)	HD(↓)	DSC(↑)	HD(↓)
			0.803 ± 0.04	3.22 ± 0.55	0.720 ± 0.05	5.10 ± 0.80
✓			0.872 ± 0.03†	1.88 ± 0.42†	0.831 ± 0.03†	2.59 ± 0.45†
	✓		0.859 ± 0.03	2.17 ± 0.45	0.809 ± 0.03	3.01 ± 0.50
		✓	0.835 ± 0.04	2.57 ± 0.50	0.738 ± 0.04	4.85 ± 0.75
✓	✓		0.898 ± 0.02*	1.71 ± 0.35*	0.849 ± 0.02*	2.11 ± 0.35*
✓		✓	0.887 ± 0.02	1.74 ± 0.38	0.836 ± 0.02	2.25 ± 0.38
	✓	✓	0.869 ± 0.02	1.90 ± 0.40	0.813 ± 0.03	2.37 ± 0.42
✓	✓	✓	**0.906 ± 0.01**	**1.33 ± 0.25**	**0.855 ± 0.01**	**2.02 ± 0.30**

**Fig 6:**
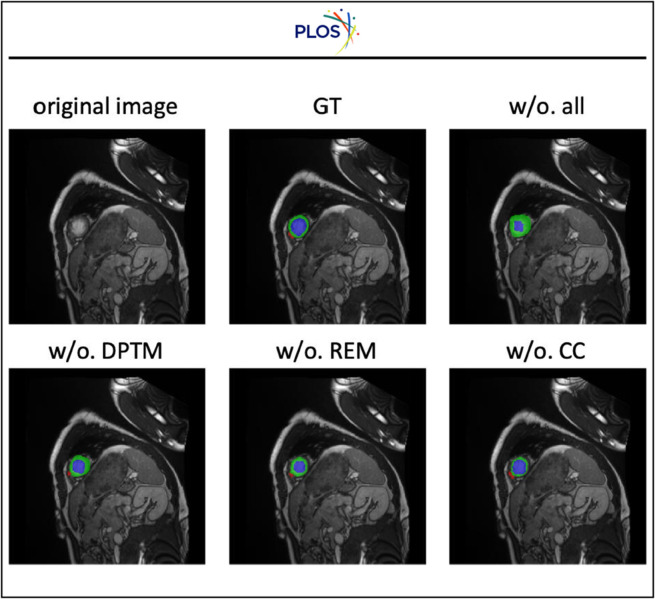
Benefits of the three proposed components dynamic pseudo-label threshold map (DPTM), robust entropy minimization (REM) and contrastive consistency (CC).

Furthermore, we visualize one example in the ACDC dataset in Fig 6 to validate the effectiveness of the three components. Without any strategies proposed in the paper, the model can not detect the small red region. When we apply one of the strategies in our paper, the model starts to detect the small red region. This fully validates the effectiveness of the three components proposed in our paper. Besides, compared with ground truth, the method without contrastive consistency can still accurately segment different regions. However, the model without the dynamic pseudo-label threshold map strategy can not segment the regions well. For example, the method without the dynamic pseudo-label threshold map strategy segments a much thicker green region compared with the ground truth and can not segment the red region well. This demonstrates the dynamic pseudo-label threshold map strategy brings more improvements compared with the other two components.

#### 4.8.2 Loss trade-off.

**Fig 7:**
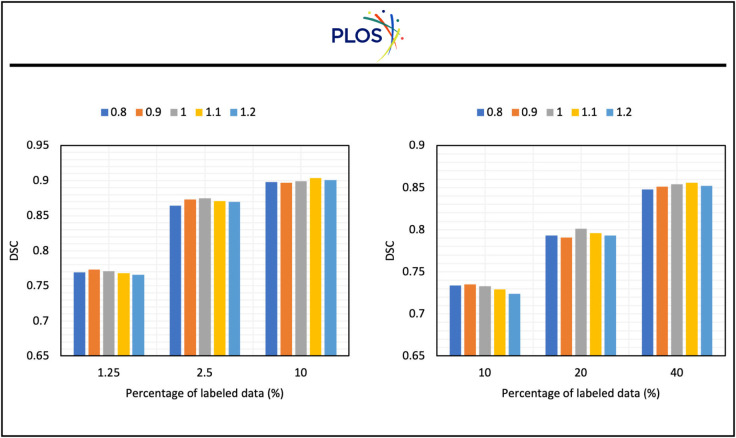
The impact of $\lambda_{1}$ on the performance of the model on two datasets.

**Fig 8:**
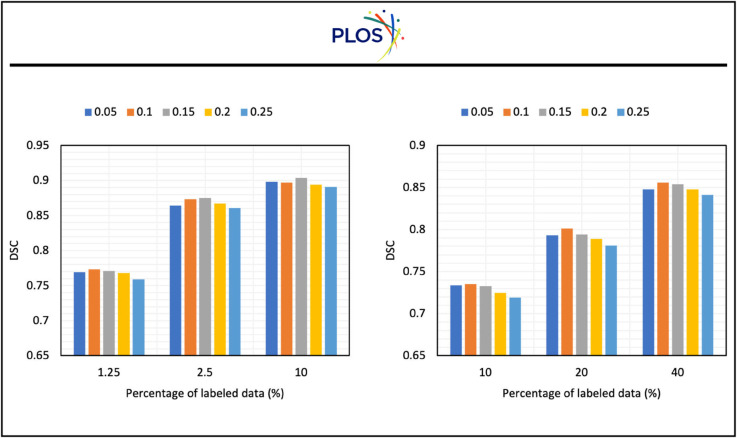
The impact of $\lambda_{2}$ on the performance of the model on two datasets.

In Sect 3.2, we introduce $\lambda_{1}$ and $\lambda_{2}$ to balance our three losses. To explore the impact of different losses on the performance of the model, we select a series of $\lambda_{1}$ and $\lambda_{2}$ and present our results in Figs 7 and 8.

Fig 7 presents the impact of $\lambda_{1}$ on the performance of the model. From the figure, we can observe that on both datasets, when the percentage of labeled data is low, a lower $\lambda_{1}$ will bring more improvements than a higher $\lambda_{1}$. Correspondingly, when the percentage of labeled data increases, the $\lambda_{1}$ that yields the best result will also increase. This is because when the percentage of labeled data is low, the model can not generate very accurate pseudo-labels, indicating that a high value of $\lambda_{1}$ will influence the training of the model. When the percentage of labeled data increases, the quality of pseudo-labels will increase, indicating that a high value $\lambda_{1}$ will make the model learn more information from the unlabeled data. Moreover, from the table, the DSC scores remain relatively stable no matter how big the $\lambda_{1}$ is. This indicates our dynamic pseudo-label threshold map strategy is robust and thus not sensitive to $\lambda_{1}$.

Fig 8 presents the impact of $\lambda_{2}$ on the performance of the model. It is evident that when $\lambda_{2}$ is around 0.1-0.15, the model has the best performance. With the increase of $\lambda_{2}$, the performance of the model will decrease. This indicates the large $\lambda_{2}$ will influence the training of the model. From the experiments, we can observe that 0.1-0.15 for $\lambda_{2}$ is a good choice.

### 4.9 Computational efficiency analysis

As described in Sect 4.2, all experiments are conducted on a Ubuntu desktop equipped with NVIDIA RTX3090 GPUs. As shown in Table 7, the training time is approximately 1.25 seconds per batch, and the inference time is 15.8 ms per image. While our method involves additional computations for dynamic thresholding and contrastive learning, the inference speed remains highly efficient for real-time clinical applications. The memory usage is approximately 20.5 GB per GPU.

**Table 7:** Computational efficiency comparison on ACDC dataset (10% labeled data).

Method	Training Time (s/batch)	Inference Time (ms/image)	Memory (GB/GPU)
Ours	1.25	15.8	20.5

The training process incorporates DPTM, robust entropy minimization, and contrastive consistency to learn robust features. While these components introduce additional calculations during the optimization phase, they are essential for extracting high-quality pseudo-labels and enforcing structural consistency from limited data.

Crucially, the proposed modules (DPTM and CC) are only active during the training phase. During the inference phase, the model operates as a standard U-Net without any auxiliary branches or thresholding operations, ensuring a rapid inference speed of 15.8 ms per image. This design makes our framework highly suitable for clinical workflows where real-time segmentation is required, justifying the reasonable computational investment during training for superior segmentation accuracy.

### 4.10 Domain adaptation analysis

To further evaluate the robustness of our method under domain shift scenarios, we conducted transfer learning experiments following PCL [57]. We pre-trained the model on the CHD dataset [64] and fine-tuned it on the MMWHS dataset with a limited number of labeled patients (*M*). As shown in Table 8, our method consistently outperforms state-of-the-art self-supervised and semi-supervised methods across different labeled data regimes, demonstrating its strong generalization capability across different scanners and protocols.

**Table 8:** Transfer learning comparison from CHD to MMWHS. All methods are pre-trained on CHD and fine-tuned on MMWHS with varying numbers of labeled patients (*M*).

Method	M=2	M=8	M=16
Random	0.232	0.769	0.834
Rotation [65]	0.247	0.768	0.850
SimCLR [50]	0.269	0.783	0.850
GCL [66]	0.262	0.805	0.851
PCL [57]	0.339	0.820	0.869
**Ours**	**0.365 ± 0.05**	**0.835 ± 0.03**	**0.878 ± 0.02**

## 5 Limitations and future work

This work presents several limitations that offer directions for future research. First, while our method performs robustly in standard low-label settings (e.g., 10%), performance stability in extremely low-label regimes (e.g., 1–2 scans) remains a challenge, as the initial pseudo-labels may be too noisy to guide the dynamic thresholding effectively. Second, the proposed framework introduces computational overhead from the dynamic threshold mapping and contrastive learning components. Although inference time remains efficient, the training resource requirements are higher than simple baselines. Third, performance degradation under severe domain shift remains an inherent challenge across different imaging centers or modalities, requiring further investigation into cross-modality generalization techniques. Furthermore, validation is currently limited to cardiac structures in MRI and CT, leaving generalization to other organs and modalities for future work. Finally, regarding clinical translation, future work should focus on integrating the model into clinical workflows to evaluate its utility in real-world diagnostic scenarios.

Building on these limitations, promising future directions include integrating transformer architectures to capture long-range dependencies and extending the method to 3D volumetric segmentation.

## 6 Conclusion

In this paper, we propose three novel strategies to address semi-supervised cardiac segmentation. Specifically, we propose a dynamic pseudo-label threshold map, robust entropy minimization and contrastive consistency from the perspective of pseudo-labeling, entropy minimization and consistency regularization, respectively. Dynamic pseudo-label threshold map strategy can generate pixel-wise, class-wise and adaptive threshold maps which can help obtain high-confident pseudo-labels. Robust entropy minimization can help adaptively optimize the model and contrastive consistency can make the model more robust. Extensive experiments are conducted to demonstrate the effectiveness of our method. Furthermore, ablation experiments are conducted for a better understanding of our method.
